# Fabrication and Characterization of Chitosan and Gelatin-Based Antimicrobial Films Incorporated with Different Essential Oils

**DOI:** 10.3390/foods13121796

**Published:** 2024-06-07

**Authors:** Laiba Asghar, Amna Sahar, Muhammad Issa Khan, Muhammad Shahid

**Affiliations:** 1 National Institute of Food Science and Technology, University of Agriculture Faisalabad, Faisalabad 38000, Pakistan; 2 Department of Food Engineering, University of Agriculture Faisalabad, Faisalabad 38000, Pakistan; 3 Department of Biochemistry, University of Agriculture Faisalabad, Faisalabad 38000, Pakistan

**Keywords:** essential oil, thyme, ginger, cumin, antimicrobial, viscosity

## Abstract

This study was performed to check the effect of different essential oils on chitosan and gelatin-based antimicrobial films. Films prepared from biopolymers contain better mechanical strength but lack in moisture barrier properties. In order to increase the moisture barrier properties of chitosan and gelatin-based films in the current research work, different essential oils, i.e., thyme, cinnamon, basil, ginger, and cumin, at varying concentrations (1.0, 1.5, and 2.0%) were incorporated. Moreover, the concentrations of glycerol (plasticizer) and emulsifier (Tween 20) were kept constant to maintain homogeneity in the research. Antimicrobial films composed of gelatin and chitosan infused with essential oils were evaluated for their physicochemical (emulsion stability, particle size, and viscosity), FT-IR, microstructural (scanning electron microscopy), moisture barrier (water vapor permeability), and antimicrobial properties (*E. coli*, *Salmonella*, and *S. aureus*). Study outcomes elucidated significant variations (*p* < 0.05) as the concentration of essential oil was increased in the film solutions. An increased concentration of essential oil (2.0%) significantly enhanced the moisture barrier properties (1.12 ± 0.03 g.mm/kPa.h.m^2^). Nevertheless, the tensile strength decreased (38.60 ± 1.4 to 31.50 ± 1.5 MPa) from 1 to 2%. The increase in essential oil concentration in the emulsion-based films also influenced their physicochemical characteristics, such as droplet size, viscosity, and emulsion stability. At lower concentrations (1.0%), films exhibited a uniform microstructure but lacked moisture barrier properties. Antimicrobial properties against *E. coli*, *Salmonella*, and *S. aureus* showed an increased inhibition effect as the concentration of essential oil was increased. Of the essential oil-based films, ginger- and basil-based films showed greater inhibition effects as compared to the other essential oils. Overall, antimicrobial films containing a 1.5% concentration of ginger and basil oil showed better results as compared to the other treatments for mechanical, moisture barrier, and antimicrobial properties, while films with a 2.0% oil concentration showed better antimicrobial and moisture barrier properties but lacked in mechanical properties. Essential oil-based antimicrobial films have prospective applications in foods, specifically in fresh and processed food items such as seafood, meat, chicken, and sausages.

## 1. Introduction

Nowadays, people are more aware and concerned about their health and the environment and demand biodegradable packaging as compared to petroleum-based materials. Numerous research investigations have been carried out in this context to find the negative effect of these petroleum-based plastics, finding that these packaging materials are destroying foods and damaging our lives. As such, the entire concept of packaging is changing to biodegradable packaging. Packaging is not only an outer covering but also a complete source of information that tells us about the temperature, safety, and freshness of the product. This is the reason organic packaging is becoming more popular in the world for the packaging of food [1].

Edible films are made up of proteins, carbohydrates, lipids, and novel ingredients that can act as antimicrobial agents and antioxidants, such as essential oils and nanoparticles, which make them capable of having more than one quality in the case of enhancing the nutrients, taste, quality, safety, and shelf life of the packaged product [2]. Among all edible films, protein-based films are attaining more acceptance due to their better mechanical properties. Zein, gluten, casein, soy protein, mung bean protein, gelatin, and collagen extracted from animals and plant-based materials are some examples of good quality proteins used for manufacturing protein-based edible packaging [3]. Gelatin is derived from collagen, and incomplete hydrolyses of collagen produce gelatin, which is a polypeptide [4]. Gelatin is viscous and has a good ability to form gels due to its high elastic modulus. It has a beta-sheet type and complex alpha-helix structure with good hydrogen bonding. Gelatin can be extracted from animal sources, mainly from bovines and pigs, but numerous health and religious issues have been reported. As such, there is a need for alternative sources, such as gelatin from chicken skin, feet, bones, fish skin, and fish bones, which are required to replace this traditional gelatin extraction to improve overall safety and acceptability [4]). Gelatin possesses several qualities that render it a viable material for food packaging. These include, among others, its affordability, ease of polymerization, biodegradability, and exceptional antibacterial and antioxidant properties [5]. Its technological functions include its gel-forming ability, water-binding ability, water vapor barrier properties, form and film-forming ability, and emulsification tendency [6].

Chitosan is derived from chitin that has been deacetylated. D-glucosamine and N-acetyl-D-glucosamine are its copolymers. Chitin is the second most prevalent polysaccharide substance, after cellulose, and because of its film-developing capabilities, along with its antioxidant and antibacterial qualities, chitosan is a suitable component for manufacturing biodegradable packaging films [7].

In a research work, Haghighi et al. [8] studied the characterization of active chitosan–gelatin blend films enriched with different essential oils (cinnamon, citronella, pink clove, nutmeg, and thyme) and concluded that films enriched with different essential oils could be used as environmentally friendly, active food packaging with antimicrobial properties.

Ginger is a very important spice with a lot of health benefits owing to its use in different medicines. It belongs to *Zingiber officinale* L. and is mostly consumed in fresh (paste or slice) and dried powder form. Its essential oil has diverse and rich phytochemical compounds, including zingiberene, curcumin, monoterpenes, camphene, geranial, and linalool, that are used as antioxidants and antimicrobial and anti-inflammatory compounds [9]. Nowadays, ginger essential oil is being used in different research studies to develop novel active and antimicrobial packaging [10].

Cumin (*Cuminum cymium* L.) has been well known since ancient times for its pharmaceutical, functional, and nutritional values, including being antimicrobial, anti-inflammatory, antihypertensive, antidiabetic, and anticancer [11]. Cumin seeds and oil are in practice used as a natural remedy against different diseases, such as cough, headache, asthma, bronchitis, etc. Generally, cumin is used as a spice or food ingredient in several products, like bakery products, breads, and salads [12]. Cumin’s crude oil exhibits better antioxidant activity owing to its higher polyunsaturated fatty acid contents; for example, of oleic, linoleic, and linolenic acids [13]. Major bioactive compounds available in black cumin include carvone, D-limonene, and p-cymene. Several studies have exhibited the antimicrobial properties of cumin essential oil versus various microbial strains [14].

Essential oil obtained from thyme (*Thymus vulgaris* L.) has shown significant medicinal properties, such as antioxidant and anti-inflammatory properties. They are used to improve the quality of food in functional food formulations, in addition to their positive effects on health. Thymol-infused film may help preserve meat, crops, and dairy products [15]. Cinnamon (*Cinnamomum verum* L.) essential oil (CEO) is a potent botanical food preservative obtained from plants. Encapsulation improves the ability to maintain the freshness of food and the stability of the CEO. Cinnamon essential oil has notable potential and unique features that make it a practical alternative for addressing various issues related to food preservation. These oils have the dual function of prolonging the shelf life of food goods and improving their taste in many refined uses [16]. 

Basil (*Ocimum basilicum* L.) has been used for many centuries as a food preservative. Basil essential oil, largely extracted from the leaf, has several functions and uses. It may be used as a preservative, an addition, or even a medicinal component in meals that include both plants and animals [17]. Basil essential oil can be used in different edible packaging films as an antimicrobial agent. Recently, a study was conducted to investigate the moisture barrier, mechanical, and antimicrobial properties of films prepared by taking basil seed oil and alginate, and the results indicated a significant effect (*p* < 0.05) among treatments. Results indicated that moisture barrier properties improved significantly and a 56.33 to 65.26% increase was recorded. Further, basil-based antimicrobial films showed a better effect against Gram positive (*Staphylococcus aureus*) and Gram negative (*E. coli*) bacteria, respectively, while significant tensile properties (2.30–2.86 MPa) were reported. Results indicated the potential application of basil oil in food packaging materials [18]. Furthermore, Amor et al. [19] prepared basil oil-based active packaging films by incorporating chitosan. Developed films were analyzed for antimicrobial properties and results indicated that basil oil-based active films significantly (*p* < 0.05) controlled the growth of all types of microbes, especially for Gram positive bacteria.

Aromatic and volatile oily liquids extracted from plant material are known as essential oils. They are made up of unique cells or groups of cells that can be found in stems, leaves, and seeds. They have antibacterial characteristics [20] and now there is an increasing focus on alternative naturally derived antimicrobials that has shifted scientific interest in these compounds [21]. The current research project is an attempt to develop chitosan and gelatin-based films incorporated with different essential oils (thyme, cinnamon, basil, ginger, and cumin) as antimicrobial agents. To check the interactions of essential oil-based films incorporated with chitosan and gelatin, their physicochemical, microstructural, mechanical, moisture barrier, and antimicrobial properties are also evaluated. The overall objective of the current study includes the development of essential oil-based edible films and the characterization of biodegradable films. 

## 2. Materials and Methods

### 2.1. Procurement of Raw Materials

Food-grade essential oils of cinnamon, thyme, basil, cumin, and ginger were procured from the Rocky Mountain Oils brand from the supermarket of Faisalabad. Moreover, chitosan and gelatin were procured from BioChem (Princeton, NJ, USA). Additionally, plasticizers, emulsifiers, and acetic acid were obtained from Sigma-Aldrich (Saint Louis, MO, USA). Likewise, distilled water was bought from a milli-Q water purification system (Millipore, Merck, Germany). 

### 2.2. Development of Antimicrobial Films

Essential oil-based films were developed through the casting method, in which samples were poured into petri dishes and kept in a hot-air oven at 40 °C for 24 h, according to the protocol given by [8]. Different types of essential oils were added in different proportions during the development of the films according to the treatment plan (Table 1). Plasticizers and emulsifiers were also added to the film-forming solutions. For the preparation of films, firstly chitosan (1 g) was dissolved in acetic acid (1%) solution (50 mL dH_2_O) under continuous stirring for 30 min at 55 °C. A gelatin solution was prepared separately by mixing it at a concentration of 2 g in distilled water (45 mL), leaving it for 15 min at 7 °C to swell, and then heating it again at 55 °C for 30 min for homogenous mixing. Then, both solutions were mixed properly, glycerol (1%) and Tween 20 (1 mL/100 mL) were added, and the total volume made to 100 mL by adding distilled water. For degassing of the essential oil-based solutions, they were kept opened for 45 min at room temperature. In the end, a different essential oil was added to each with continuous stirring for 30 min. 

All the experiments were performed in triplicates. 

### 2.3. Characterization of Essential Oil-Based Films

Essential oil-based antimicrobial films with gelatin and chitosan as base materials were characterized for their different physicochemical, microstructural, mechanical, moisture barrier, and antimicrobial properties. 

#### 2.3.1. Emulsion Stability

The essential oil-based film solutions’ stability was determined according to the protocol of [22]. The developed solution (10 mL) was taken in an airtight screwcap test tube and kept at 25 °C for 3 days. The initial and final volume of the film solutions was measured and changes in phase separation were noted. The stability of the films was computed by using the following formula:

$$S=\frac{h_{o}\;-h_{t}}{h_{o}}\times 100$$

S = Stability.

h_o_ = Initial volume of emulsion.

h_t_ = Final volume of the emulsion after 72 h.

#### 2.3.2. Particle Size

The essential oil-based film emulsions’ particle size was measured by using a laser particle size analyzer according to the procedure of [23]. For measuring particle size, a polydispersity distribution model from Schultz was used. Samples were captured and their obscuration was measured, which is based upon the lost power of laser light while it is passed through the film emulsion. The readings for particle size of the emulsion samples were recorded after 2 to 3 min of dispersion and the mean values were taken from the average of triplicate replications. 

#### 2.3.3. Viscosity

The essential oil-based coating solutions’ viscosity was checked through a rheometer according to the protocol described by [24]. The coating solution (33 mL) was taken into the concentrical cylinder probe at 25 °C and measurement was taken at a shear rate of 0–300 s^−1^. Before measuring the viscosity of the coating solutions, it was ensured that the temperature of the rheometer and the samples was the same while running samples for analysis. 

#### 2.3.4. Fourier Transform Infrared Spectroscopy (FTIR)

FTIR spectra were collected in the mid-infrared (MIR) range (2.5–50 µm) by using ZnSe (zinc selenide) crystal with an ATR (attenuated total reflectance) accessory on a FTIR spectrophotometer to assess the interactions between gelatin, glycerol, and essential oils according to the method of [25]. The scanning wavelengths of different film samples were taken at the range of 4000 to 600 cm^−1^ at the resolution spectra of 4 cm^−1^. Measurements of FTIR were taken at room temperature (25 °C) and data were recorded in triplicate. The peaks of the different treatments of films from T_1_ to T_15_ were identified through software and assigned through literature values.

#### 2.3.5. Mechanical Properties of the Essential Oil-Based Films

The mechanical properties of the essential oil-based films were assessed by using a Universal Testing Machine (HD-B607-2, Haida International Equipment, Dongguan, China) by following the method given by [26]. The prepared films were cut into 15 mm width and 45 mm length pieces and kept at ambient temperature and 70% humidity overnight. The prepared samples were fixed in loading frames and the length of the gauge was fixed at 50 mm and exposed to 50 N force until the breaking of the film. However, the speed of each film’s stretching was kept at 100 mm/min. 

#### 2.3.6. Water Vapor Permeability

The essential oil-based films’ water vapor permeability was measured to assess the moisture barrier properties of the films by using the standard method of [27]. In this regard, films were applied to permeability cups containing 10 mL of deionized water and stored at room temperature (25 °C) and 60% humidity level. Likewise, the change in weight was calculated over 12 h, measured after each 1 h interval. The following equation was used to determine the water vapor permeability of the films.

$$\mathrm{WVP}=\frac{\mathrm{Slope}\;\times \;L}{A\;\times \;∆P}$$

L = Film average thickness (m).

A = Moisture transfer area of the cup (m^2^).

∆P = Difference of partial water vapor pressure.

Slope = Constant for weight change vs. time.

#### 2.3.7. Antimicrobial Activity of Films

The essential oil-based films’ antimicrobial activity against different microbial strains (*E. coli* (O157:H7 ATCC 35150), *Salmonella* (*Salmonella enterica* ATCC 35664)*,* and *Staphylococcus* (*S. aureus* ATCC 23235)) was assessed by using the protocol as described by [7]. The strains of (*E. coli* (O157:H7 ATCC 35150)*, Salmonella* (*Salmonella enterica* ATCC 35664)*,* and *Staphylococcus* (*S. aureus* ATCC 23235)) were bought from the Microbiologics inc. Saint Cloud, Minnesota, USA. In this regard, 0.9 g sodium chloride was mixed properly in 10 mL distilled water to prepare a 0.9% saline solution. Around 2.8 g of nutrient agar was mixed in 100 mL of deionized water. The nutrient media (Merck Millipore, Burlington, MA, USA) flask was kept in a heated water bath to reach 90 °C. Furthermore, nutrient agar plates were inoculated. After, the heated media was cooled down to about 45 °C for 15 min before inoculation. In the end, the already-prepared films stored in a desiccator at room temperature were cut into 12 mm parts and spread in the inoculum of *Escherichia coli, Salmonella,* and *Staphylococcus aureus* on solidified petri dishes aseptically and incubated at 37 ± 0.5 °C for 24 h. The inhibition zone diameters were measured by using a metric ruler and the entire area of the clear zone was calculated for *E. coli*, *Salmonella*, and *S. aureus*. Analysis was performed in triplicate to check the effectiveness of the developed films against selected strains of microbes, as given above. Control treatments were also performed for each microbe without adding film samples. 

### 2.4. Statistical Analysis

All the obtained data from different parameters of essential oil-based films were subjected to factorial statistical analysis via ANOVA to check the level of significance. The statistical analysis was performed through Origin-Pro 2021b 9.85 (Origin Lab Co., Northampton, MA, USA). Furthermore, the post-hoc comparison of means was performed by using Tukey’s honest significant difference (HSD) test [28].

## 3. Results and Discussion

### 3.1. Emulsion Stability

Mean squares for the emulsion stability of essential oil-based films are illustrated in Table 2, which demonstrates significant differences (*p* ˂ 0.05) across the various treatments. In essential oil-based film treatments, the highest mean values for film emulsion stability were observed in T3 (93.40 ± 3%), T6 (95.70 ± 4.3%), T9 (90.67 ± 6.7%), T12 (92.53 ± 2.50%), followed by T15 (95.66 ± 3.30%) for thyme, cinnamon, basil, ginger, and cumin, respectively. Nevertheless, the minimum value for emulsion stability among all treatments was noticed in T7 (85.30 ± 2.61%). The remaining treatments showed significant variations among different essential oils as changes in concentration resulted in increased emulsion stability, respectively (Table 3). Furthermore, the overall composition and particle size of the film solution also influenced the emulsion stability significantly. 

The emulsion stability of films is influenced by different factors, including the films’ composition, formulation, type, and concentration of essential oils, as well as the choice of emulsifier used during development. Emulsifiers play a vital role in stabilizing emulsions by reducing the pressures acting at the interface between two immiscible phases, such as water and oil. Emulsifiers contributed to the development of an oil-in-water emulsion film solution that effectively inhibits the aggregation of dispersed lipid particles [22,29]. examined the emulsion stability of sodium alginate-based film with clove essential oil. They made clove essential oil (1.0%) films using chitosan (2.5%) and emulsifier (Tween 80). The combination of a lower essential oil content, homogenizer speed, and duration produced 2.3 µm droplets with over 90% emulsion stability. However, emulsion stability showed substantial alterations (*p* < 0.05) after 4 weeks of storage at ambient temperature. The results of this research are aligned with the current study outcomes, where all the developed films showed an emulsion stability > 85.0%, which is appropriate for the development of edible films.

### 3.2. Particle Size

Mean squares for the particle size of the essential oil-based emulsions are demonstrated in Table 2, revealing significant (*p* ˂ 0.05) differences between treatments. In essential oil-based film treatments, the smallest particle size was observed in T1 (2.41 ± 0.41 µm), T4 (1.40 ± 0.02 µm), T7 (2.17 ± 0.03 µm), T10 (3.48 ± 0.02 µm) and T13 (5.41 ± 0.02 µm) for thyme, cinnamon, basil, ginger, and cumin, respectively. However, the smallest size particles were recorded in T4 (1.40 ± 0.02 µm), preceded by the T7 treatment (2.17 ± 0.03 µm), among the cinnamon and basil treatments, respectively. The other essential oil-based films treatments demonstrated an increased particle size as the concentration of essential oils increased in the film preparation, as given in Table 3. Conclusively, it is obvious from the findings that particle size is directly affected by essential oil concentrations during film formulation.

The outcomes reported in the current study are in accordance with the results reported by [30]. They developed chitosan-based films incorporated with thyme essential oil. A larger particle size was noticed in the treatments as the concentration of essential oil increased. Similarly, in the current research, the particle size also increased when the concentration of essential oil increased (2%) in the film-forming solution. There are some other factors that affect particle size, like the homogenization process, as the time–temperature combination of the homogenizer significantly affects (*p* < 0.05) particle size. Low speed and less time results in larger particles in the film-forming solution, which ultimately results in roughly structured films. 

### 3.3. Viscosity

Table 2 shows the mean squares for the viscosity of the essential oil-based films, revealing non-significant (*p* > 0.05) changes among the treatments. In the essential oil-based film treatments, the maximum value for viscosity was recorded in T9 (0.31 ± 0.09) Pas followed by T8 at 0.28 ± 0.03 Pas, respectively, from the same group of essential oil (basil). Nevertheless, among the other treatments, the maximum value for viscosity was recognized in T3 (0.17 ± 0.66 Pas), T6 (0.24 ± 0.06 Pas), T12 (0.16 ± 0.05 Pas), and T15 (0.17 ± 0.66 Pas) for thyme, cinnamon, ginger, and cumin, respectively. All other treatments of essential oil-based films (Table 3) showed an increase in viscosity as the concentration of essential oils increased. In general, it can be stated that an increase in the concentration of the essential oils in the treatments resulted in a substantial improvement in the viscosity of the film solutions.

The findings of the present study are also aligned with the outcomes of the earlier study conducted by [31]. They developed chitosan-based films in combination with thyme essential oil. Their study’s results depicted significant (*p* < 0.05) variations among treatments as concentrations used, types of ingredients, and processing conditions employed during the development of the film-forming emulsion. Different values were noticed for the viscosity of the film emulsions, and these differences were reported due to the change in concentration of the essential oil used and processing conditions employed. There are some factors that affect the viscosity of the film-forming solution, including the concentration of solid particles, mixing time, homogenizer speed, and most importantly the cross-linking reagent used in the film-forming solution.

### 3.4. Fourier Transform Infrared Spectroscopy (FTIR) 

FTIR spectra are used to check the intermolecular forces among ingredients used for the development of different products. In the film-forming structures, the FTIR spectra wavelength remained constant, but the height of the peak increased significantly with the increase in the concentration of essential oils in the different treatments. 

**Figure 1:**
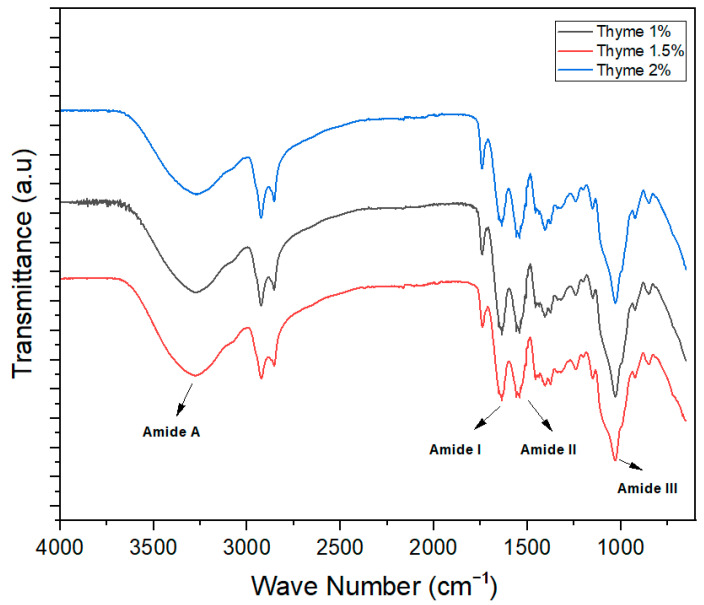
FTIR spectra of thyme essential oil-based edible films.

Figure 1 shows the FTIR spectra of thyme oil-incorporated edible films for 1%, 1.5%, and 2% oil concentration in the wavelength range of 4000–400 cm^−1^. Since the grade of chitosan used in the present research was C80% decaylated, the C=O stretching (amide I) peak at 1625 cm^−1^, C-N stretching (amide II) peak at 1510 cm^−1^, peak for amide III at 1170 cm^−1^_,_ and N-H stretch (amide A) peak at 3375 cm^−1^ were observed. 

**Figure 2:**
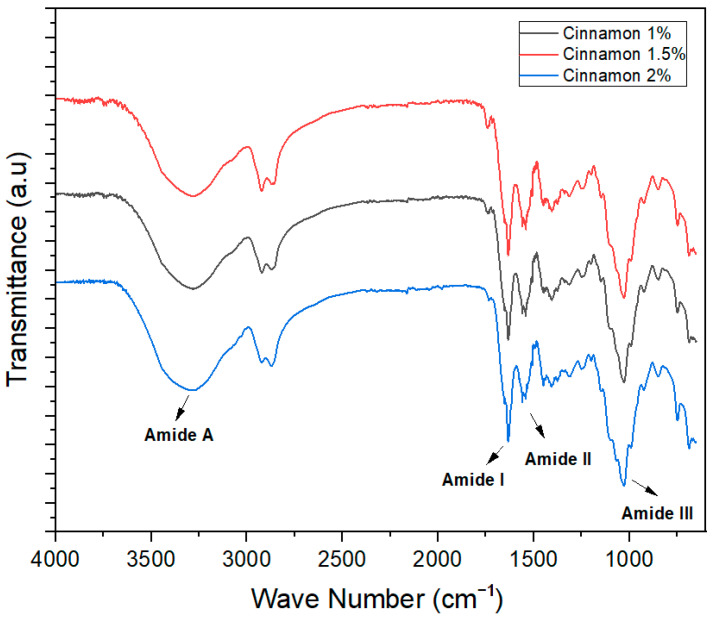
FTIR spectra of cinnamon essential oil-based edible films.

The FTIR spectra of cinnamon oil-incorporated edible films at different oil concentrations were studied in the wavelength range of 4000–400 cm^−1^. The band around 1675 cm^−1^ was related to C=O stretching (amide I). Oxidation of essential oil increased the intensity of this band. The C-N stretching (amide II) peak at 1460 cm^−1^, peak for amide III at 1155 cm^−1^_,_ and N-H stretching (O-H stretching) amide A peak at 3312 cm^−1^ were observed (Figure 2). Previously, a study by Haghighi et al. [32] developed and characterized fish gelatin-based films incorporated with cinnamon essential oil at different concentrations (2%, 4%, and 6%) and showed similar results that align with those of the current study. 

**Figure 3:**
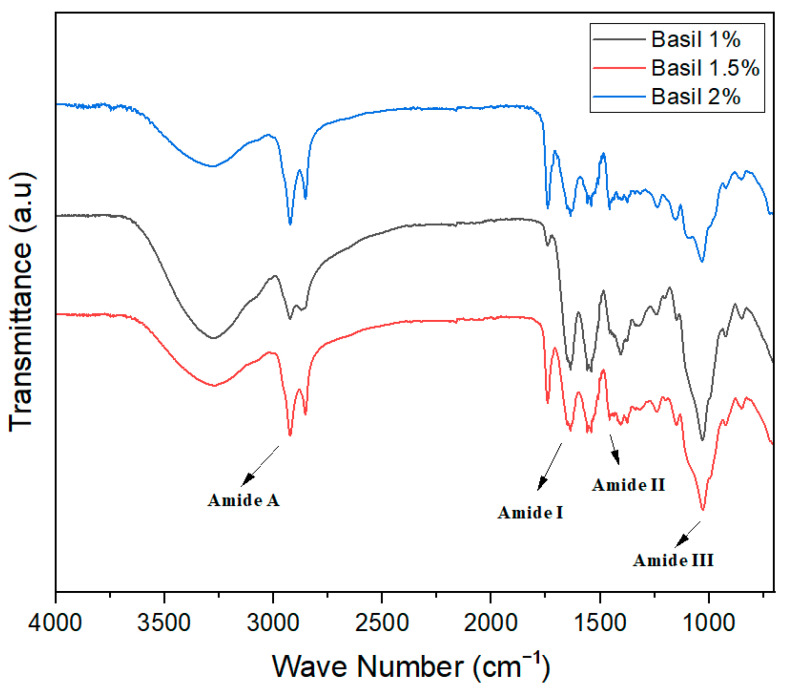
FTIR spectra of basil essential oil-based edible films.

For basil essential oil-based edible films, a prominent amid A band at 2990 cm^−1^ in the chitosan spectrum correlated to N-H and O-H stretching. The bands assigned to C-O stretching (amide I), C-N stretching (amide III), and C-H stretching (amide II) appeared at 1690 cm^−1^, 1122 cm^−1^_,_ and 1470 cm^−1^_,_ respectively, confirming the presence of N-acetyl groups (Figure 3). Likewise, a research study was conducted by Hemalatha et al. [33], in which they developed and characterized chitosan-based films with basil essential oil and determined their acceptability for food packaging. 

**Figure 4:**
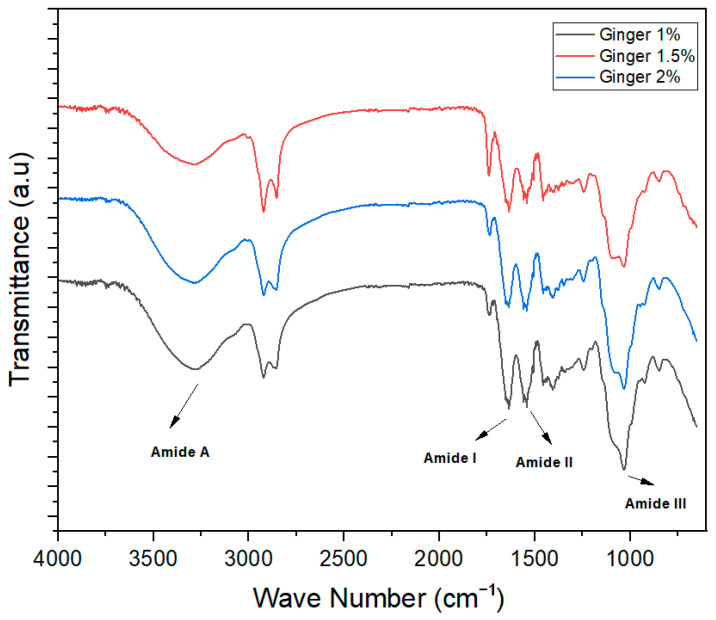
FTIR spectra of ginger essential oil-based edible films.

Figure 4 shows the FTIR spectra of ginger oil-incorporated edible films for 1%, 1.5% and 2% oil concentration in the wavelength range of 4000–400 cm^−1^. The C=O stretching (amide I) peak at 1715 cm^−1^, C-N stretching (amide II) peak at 1610 cm^−1^, peak for amide III at 1125 cm^−1^, and N-H stretch (amide A) peak at 3350 cm^−1^ were observed. Similar results were reported by a research study conducted by [34] which indicated the existence of a C=O bond (amide 1) and in which FTIR spectra showed a peak at 1625 cm^−1^, respectively. In the developed films, C-N and N-H bonding were reported among 1602 cm^−1^ to 3500 cm^−1^, respectively. 

**Figure 5:**
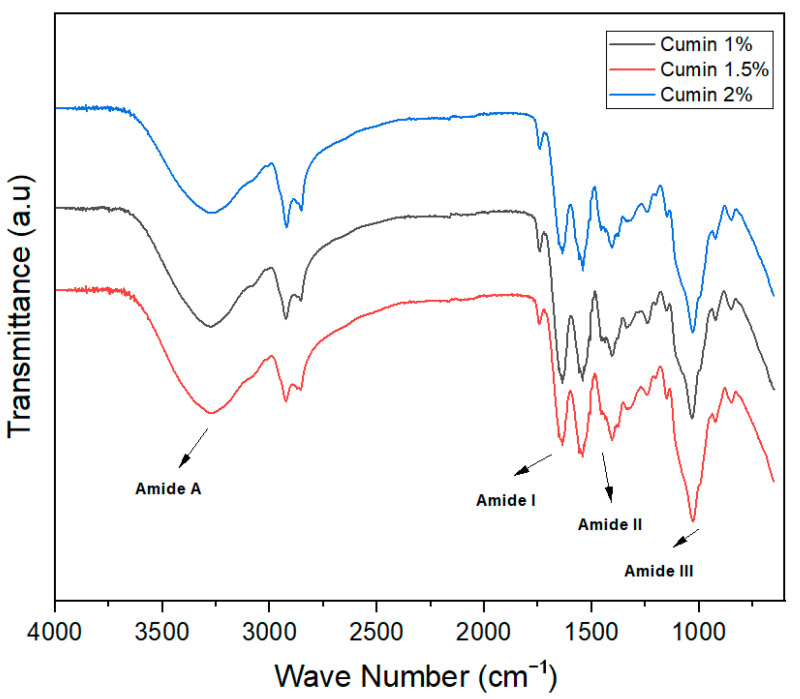
FTIR spectra of cumin essential oil-based edible films.

Lastly, for cumin essential oil-based edible films, band peaks of 1109 cm^−1^, 1480 cm^−1^, 1710 cm^−1^, and 3350 cm^−1^ were found in each treatment. The peak at 1109 cm^−1^ was due to the O-H bond, while those at 1480 cm^−1^ and 3350 cm^−1^ were due to C-N stretching and C-H stretching, respectively. The peak around 1710 cm^−1^ was associated with stretching vibration of the C=O group (Figure 5). Previously, Ref. [35] conducted a study and reported similar outcomes. However, there is a difference in the wavelength number reported in that study as compared to that in the current study, due to the different polymers and the different concentration of cumin oil used in the film formation, respectively. The reported results are aligned with the outcomes of the current study, as similar results are reported for the bonding and wavelength numbering with a slight difference in all treatments.

### 3.5. Mechanical Properties

Mean squares for the mechanical properties, including tensile strength, elongation at breakage, and Young’s modulus, of the essential oil-based films are given in Table 4 which depicted statistically significant (*p* ˂ 0.05) differences among treatments. Essential oil-based films with gelatin and chitosan as the base materials showed better mechanical properties and significant differences were observed among treatments. In essential oil-based films, the maximum mean values of tensile strength (Table 5) were observed in T1 (37.50 Mpa), T4 (38.60 Mpa), T7 (29.60 Mpa), T10 (38.10 Mpa), and T13 (34.60 Mpa) for thyme, cinnamon, basil, ginger, and cumin, respectively. However, the minimum value for tensile strength among all treatments was recorded in T9 (24.80 Mpa). The results showed that there was a decrease in the tensile strength of the essential oil-based film samples as the concentration of essential oil increased. This means that the reduction in tensile strength was proportional to the increase in essential oil concentration. Overall, among films formulated with thyme, cinnamon, basil, ginger, and cumin essential oils, those composed of cinnamon and ginger essential oils showed the highest tensile strength. Conversely, films developed with a concentration of 2.0% basil essential oil demonstrated the lowest tensile strength, as compared to 1% basil essential oil. Hence, the concentration of the biopolymers used directly affects the tensile strength due to their role in promoting intermolecular interactions which increase the tensile strength. 

On the contrary, the elongation at breakage increased significantly (*p* < 0.05) as the concentration of essential oils increased in the film-forming matrix. The maximum value for elongation at breakage was observed in T9 (135 ± 4.0%) followed by T15 as 131.3 ± 3.1%, respectively. However, the lowest value was recorded in the T10 treatment (36 ± 2%) from the ginger essential oil-based film. Moreover, in other treatments, elongation at breakage showed an increasing effect as the concentration of oils increased. In a nutshell, the basil and cumin essential oil-based films showed the maximum elongation at breakage, while the lowest value was recorded for the ginger essential oil-based film. 

Similarly, the maximum values for Young’s modulus were recorded in T3 (195 ± 2.8 Mpa), T6 (187 ± 3 Mpa), T9 (388 ± 4 Mpa), T12 (182 ± 4 Mpa), and T15 (346 ± 4 Mpa) for thyme, cinnamon, basil, ginger, and cumin, respectively. However, the lowest value for Young’s modulus was reported for the T4 treatment (138 ± 3 Mpa). However, in the remaining treatments, a significantly increasing trend was noticed as the concentration of essential oil increased in the film. For elongation at breakage, the maximum values were recorded in T3 (54.4 ± 1.6%), T6 (106 ± 4%), T9 (135 ± 4%), T12 (58 ± 3.3%), and T15 (131 ± 3.1%) for thyme, cinnamon, basil, ginger, and cumin, respectively. The results showed that the basil essential oil-based films showed the maximum values for elongation at breakage and Young’s modulus. However, the concentration of essential oils did not show the same trend for Young’s modulus and elongation at breakage as for tensile strength. 

Typically, the incorporation of lipid constituents, such as fats or oils, into the structure of the film leads to a reduction in mechanical strength and an increase in moisture resistance. In addition, the lipid compound facilitates the formation of molecular chains with a more compact structure, which leads to reduced mechanical strength. However, in terms of mechanical properties, the results of the current study were comparable to those observed in films prepared from animal or plant proteins ([26,36]). The chemical structures of the compounds used in film-forming solutions significantly influence the mechanical properties of developed films. As chemical structure influences the rheology of film-forming solutions, it directly correlated with the mechanical strength of the developed films. The structure of the different chemicals used in the film-forming solution significantly influences the molecular chain formation that helps in improving the tensile strength and elasticity of the film. 

### 3.6. Moisture Barrier Properties

#### Water Vapor Permeability

The mean square values for the water vapor permeability of the essential oil-based films showed a significant (*p* ˂ 0.05) effect among different treatments (Table 6). In the developed films, the highest values for water vapor permeability were observed in T1 (3.16 ± 0.05 g.mm/kPa.h.m^2^), T4 (1.90 ± 0.04 g.mm/kPa.h.m^2^), T7 (1.91 ± 0.04 g.mm/kPa.h.m^2^), T10 (2.60 ± 0.04 g.mm/kPa.h.m^2^), and T13 (2.42 ± 0.08 g.mm/kPa.h.m^2^) for thyme, cinnamon, basil, ginger, and cumin, respectively. However, the lowest value for water vapor permeability was observed in T12 (1.12 ± 0.03 g.mm/kPa.h.m^2^) (Table 7). Other treatments of essential oil-based films showed an increasing effect on water vapor permeability as the concentration of essential oil increased. Among thyme, cinnamon, basil, ginger, and cumin essential oil-based films, ginger-based films showed better results against water penetration into the films and the lowest value was recorded at a 2% concentration of oil. The water vapor permeability of the films improved with the increase in essential oil concentration. 

The use of essential oils in the film-forming solutions to bind the films significantly enhanced their permeability to water vapor. Conversely, the water vapor permeability of films developed from biopolymers containing lipids was observed to be lower in comparison to those developed from a monolayer of lipids. The enhanced water vapor permeability of monolayer-based films can be attributed to a more effective mechanism in comparison to heterogeneous biopolymer-based films containing essential oils. At concentrations between 1.0% and 1.5%, the moisture transfer characteristics of films containing essential oils do not exhibit significant variation. In the film-forming structure, however, substantial variations are undoubtedly apparent at higher concentrations of lipid constituents [37]. 

A similar study was conducted by Alexandre et al. [38], in which they prepared gelatin-based films in combination with activated nanoemulsions of ginger essential oil (GEO) for food packaging applications. Ginger essential oil-based films were developed by taking different concentrations of oil at 1, 3, and 5%, respectively. The developed films were investigated for their rheological, physiochemical, mechanical, and moisture barrier properties. The maximum water vapor permeability in the ginger essential oil-based film showed 0.27 ± 0.02 g.mm/kPa.h.m^2^. However, in the current study, water vapor permeability was measured at 1.12 ± 0.03 g.mm/kPa.h.m^2^. The change in water vapor permeability is due to the change in the concentrations of the film-forming solutions. Additionally, significant variations (*p* < 0.05) in water vapor permeability were identified in the film-forming solutions due to the different concentrations of essential oil being used.

### 3.7. Antimicrobial Properties

Table 8 shows the mean squares for the antimicrobial properties of the essential oil-based films and illustrates the significant (*p* ˂ 0.05) variations among treatments. The prepared essential oil-based films were analyzed against *Salmonella*, *Escherichia coli* (*E. coli*), and *Staphylococcus aureus* (*S. aureus*). The films against *Salmonella* showed the maximum inhibition zones in treatments T_3_ (14.60 ± 0.4 mm), T_6_ (12.60 ± 0.4 mm), T_9_ (14.50 ± 0.5 mm), T_12_ (14.80 ± 0.3 mm), and T_15_ (12.80 ± 0.2 mm) for thyme, cinnamon, basil, ginger, and cumin, respectively. However, the minimum inhibition value was observed in T_4_ as 8.70 ± 0.3 mm (Table 9). Furthermore, the other treatments for antimicrobial properties against *Salmonella* showed increasing an inhibition effect as the concentration of essential oil increased in the film-forming solution. 

The antimicrobial properties of essential oil-based films against *E. coli* showed that the maximum values for the inhibition zone were recorded in T_3_ (12.07 ± 0.3 mm), T_6_ (14.91 ± 0.2 mm), T_9_ (14.70 ± 0.4 mm), T_12_ (15.30 ± 0.2 mm), and T_15_ (13.93 ± 0.5 mm) for thyme, cinnamon, basil, ginger, and cumin, respectively. Nevertheless, the lowest value for the inhibition effect against *E. coli* was recorded in T_1_ (8.50 ± 0.1 mm). For the other treatments of essential oil-based films, an increasing inhibition zone was reported as the concentration of essential oils increased from 1.0% to 2.0%, respectively. Similarly, in the case of *S. aureus*, the maximum inhibition zones were measured in T_3_ (11.37 ± 0.63 mm), T_6_ (16.10 ± 0.9 mm), T_9_ (15.10 ± 0.9 mm), T_12_ (15.22 ± 0.78 mm), and T_15_ (14.60 ± 0.4 mm) for thyme, cinnamon, basil, ginger, and cumin, respectively. On the contrary, the lowest value for the inhibition zone was noticed in T_1_ and preceded by T_2_, i.e., 7.96 ± 0.14 and 9.24 ± 0.25 mm, respectively. A similar study was conducted by [39] who prepared and characterized Ecklonia cava alginate-based films in combination with different concentrations of cinnamon leaf and bark essential oils. Their study’s results also showed that the essential oils from cinnamon bark and leaves showed more antimicrobial properties against *E. coli*, *Salmonella*, *S. aureus*, and *Listeria monocytogens*, respectively. Another study was conducted by [40] to check the antimicrobial properties of chitosan, gum arabic, and polyvinyl alcohol-based composite films incorporated with different essential oils i.e., black pepper and ginger essential oils. The study outcomes reported that the black pepper and ginger essential oils used in the chitosan/gum arabic/polyvinyl alcohol-based films showed higher antimicrobial properties against various microbial species, including Staphylococcus aureus, *Salmonella*, Bacillus cereus, and Escherichia coli. 

Recently, Gedikoğlu [41] conducted a study to investigate the effect of *Thymus vulgaris* and *Thymbra spicata* essential oils in combination with pectin-based edible films for the shelf stability of sliced bolognas. The study outcomes are in similarity with those of this study, as an increasing trend was noticed for *Salmonella*, lactic acid bacteria, and yeast and mold count during storage. The microbial count for *Salmonella* was higher as compared to the current study. It showed that the essential oils used in the current study expressed better antimicrobial properties. However, essential oil-based film-coated meat better retained the increase in microbial count in all samples. A similar effect was recorded for the remaining treatments and the results showed that with the increase in essential oil concentration, the inhibition zone against microbe growth also increased significantly. Conclusively, of the essential oil-based films, the ginger- and basil-based films showed greater inhibition effects as compared to the other essential oils. However, thyme essential oil showed minimum antimicrobial properties against *E. coli* and *S. aureus*, respectively. Further, cinnamon showed minimum antimicrobial properties against *Salmonella* [42]). 

## 4. Conclusions

This study’s outcomes showed that both biopolymers and lipid-based compounds, especially essential oils, have shown their potential for the development of antimicrobial packaging films. Essential oil-based films were developed by utilizing different concentrations (0.5, 1, 1.5%) of essential oils (thyme, cinnamon, basil, ginger, and cumin). Results indicated that an increase in essential oil concentration significantly (*p* < 0.05) increased the water vapor permeability of chitosan and gelatin-based antimicrobial films. However, the concentration of essential oil in the film-forming matrix also influenced the tensile properties of the films, while the addition of the appropriate concentration of essential oils in the film-forming solution certainly affected tensile and moisture barrier properties (*p* < 0.05). In this regard, the films with a 1.5% concentration of ginger and basil oil demonstrated good mechanical, moisture barrier, and antimicrobial properties as compared to other films. Furthermore, essential oil-incorporated films showed better antimicrobial properties against *E. coli*, *Salmonella*, and *S. aureus* growth that ultimately enhance their application on food items and improve shelf stability. Thus, if essential oil-incorporated antimicrobial films are developed with suitable concentrations of film-forming constituents, this will not only improve their mechanical, moisture barrier, and antimicrobial properties but may have the potential to improve the shelf stability of various foods, especially meat, sausages, chicken, and seafood items. 

## Figures and Tables

**Table 1:** Development of essential oil-based antimicrobial films.

Treatments	Essential Oils (EOs)				
	Thyme (%)	Cinnamon (%)	Basil (%)	Ginger (%)	Cumin (%)
T_1_	1	-	-	-	-
T_2_	1.5	-	-	-	-
T_3_	2	-	-	-	-
T_4_	-	1	-	-	-
T_5_	-	1.5	-	-	-
T_6_	-	2	-	-	-
T_7_	-	-	1	-	-
T_8_	-	-	1.5	-	-
T_9_	-	-	2	-	-
T_10_	-	-	-	1	-
T_11_	-	-	-	1.5	-
T_12_	-	-	-	2	-
T_13_	-	-	-	-	1
T_14_	-	-	-	-	1.5
T_15_	-	-	-	-	2

**Table 2:** Mean squares for emulsion stability, particle size, and viscosity of essential oil-based edible films.

SOV	df	Emulsion Stability	Particle Size	Viscosity
Treatments	14	2.66 *	426.13 **	1.32 ^NS^
Error	30	341.447	0.948	0.35640
Total	44	765.463	189.535	0.57652

** = Highly significant (*p* < 0.01), * = Significant (*p* < 0.05), NS = Non-significant (*p* > 0.05), while df and SOV stand for Degree of Freedom and Source of Variation, respectively.

**Table 3:** Means for emulsion stability, particle size, and viscosity of essential oil-based edible films.

Treatments	Emulsion Stability (%)	Particle Size (µm)	Viscosity (Pas)
T_1_	87.80 ± 2.2 ^ab^	2.41 ± 0.41 ^gh^	0.12 ± 0.05 ^a^
T_2_	90.10 ± 2.9 ^ab^	3.79 ± 0.02 ^f^	0.13 ± 0.06 ^a^
T_3_	93.40 ± 3 ^ab^	4.50 ± 0.03 ^e^	0.17 ± 0.06 ^a^
T_4_	91.26 ± 2.7 ^ab^	1.40 ± 0.02 ^i^	0.17 ± 0.03 ^a^
T_5_	94.10 ± 2.9 ^ab^	2.91 ± 0.04 ^g^	0.20 ± 0.04 ^a^
T_6_	95.70 ± 3.3 ^a^	3.84 ± 0.01 ^f^	0.24 ± 0.06 ^a^
T_7_	85.30 ± 2.61 ^b^	2.17 ± 0.03 ^h^	0.25 ± 0.03 ^a^
T_8_	87.80 ± 2.65 ^ab^	4.57 ± 0.02 ^e^	0.28 ± 0.03 ^a^
T_9_	90.67 ± 2.3 ^ab^	6.81 ± 0.04 ^c^	0.31 ± 0.09 ^a^
T10	87.10 ± 2.9 ^ab^	3.48 ± 0.02 ^f^	0.09 ± 0.07 ^a^
T11	90.63 ± 3.21 ^ab^	5.79 ± 0.02 ^d^	0.13 ± 0.06 ^a^
T12	92.53 ± 2.50 ^ab^	7.75 ± 0.04 ^b^	0.16 ± 0.05 ^a^
T13	91.60 ± 2.30 ^ab^	5.41 ± 0.02 ^d^	0.13 ± 0.04 ^a^
T14	93.66 ± 2.40 ^ab^	6.93 ± 0.05 ^c^	0.15 ± 0.07 ^a^
T15	95.66 ± 3.30 ^a^	8.39 ± 0.03 ^a^	0.19 ± 0.06 ^a^

Similar letters in the columns indicate a non-significant (*p* > 0.05) effect among treatments. T_1_ = Thyme EO 1.0 g, T_2_ = Thyme EO 1.5 g, T_3_ = Thyme EO 2.0 g, T_4_ = Cinnamon EO 1.0 g, T_5_ = Cinnamon EO 1.5 g, T_6_ = Cinnamon EO 2.0 g, T_7_ = Basil EO 1.0 g, T_8_ = Basil EO 1.5 g, T_9_ = Basil EO 2.0 g, T_10_ = Ginger EO 1.0 g, T_11_ = Ginger EO 1.5 g, T_12_ = Ginger EO 2.0 g T_13_ = Cumin EO 1.0 g, T_14_ = Cumin EO 1.5 g, T_15_ = Cumin EO 2.0 g.

**Table 4:** Mean squares for mechanical properties of essential oil-based edible films.

SOV	df	Tensile Strength	Elongation at Breakage	Young’s Modulus
Treatments	14	24.48 **	464.71 **	1286.76 **
Error	30	56.725	266.4	477
Total	44	704.832	58,031.4	286,961

** = Highly significant (*p* < 0.01). While, df and SOV stands for Degree of Freedom and Source of Variation.

**Table 5:** Means for mechanical properties of essential oil-based edible films.

Treatments	Tensile Strength (Mpa)	Elongation at Breakage (%)	Young’s Modulus (Mpa)
T_1_	37.50 ^ab^	38.40 ± 1.6 ^h^	176 ± 2.9 ^h^
T_2_	35.20 ^a–c^	44.68 ± 2 ^h^	188 ± 3.5 ^gh^
T_3_	34.68 ^a–c^	54.4 ± 1.6 ^g^	195 ± 2.8 ^f^
T_4_	38.60 ^a^	81 ± 2.4 ^f^	138 ± 3 ^k^
T_5_	34.20 ^bc^	93 ± 3.5 ^e^	151 ± 2.4 ^ij^
T_6_	31.50 ^c-e^	106 ± 2.4 ^d^	187 ± 3 ^gh^
T_7_	29.60 ^de^	120 ± 3.7 ^bc^	283 ± 3.6 ^d^
T_8_	27.90 ^ef^	128 ± 3 ^ab^	365 ± 2.5 ^b^
T_9_	24.80 ^f^	135 ± 2.4 ^a^	388 ± 3.4 ^a^
T10	38.10 ^ab^	36 ± 2 ^h^	148 ± 3.5 ^jk^
T11	34.80 ^a–c^	43.7 ± 1.7 ^h^	160 ± 3 ^i^
T12	31.90 ^c–e^	58 ± 3.3 ^g^	182 ± 3.4 ^h^
T13	34.60 ^a–c^	103.3 ± 2.2 ^d^	210 ± 2.5 ^f^
T14	32.10 ^cd^	118 ± 3 ^c^	231 ± 2.8 ^e^
T15	29.20 ^de^	131.3 ± 3.1 ^a^	346 ± 3.4 ^c^

Similar letters in the columns indicate a non-significant (*p* > 0.05) effect among treatments. T_1_ = Thyme EO 1.0 g, T_2_ = Thyme EO 1.5 g, T_3_ = Thyme EO 2.0 g, T_4_ = Cinnamon EO 1.0 g, T_5_ = Cinnamon EO 1.5 g, T_6_ = Cinnamon EO 2.0 g, T_7_ = Basil EO 1.0 g, T_8_ = Basil EO 1.5 g, T_9_ = Basil EO 2.0 g, T_10_ = Ginger EO 1.0 g, T_11_ = Ginger EO 1.5 g, T_12_ = Ginger EO 2.0 g T_13_ = Cumin EO 1.0 g, T_14_ = Cumin EO 1.5 g, T_15_ = Cumin EO 2.0 g.

**Table 6:** Mean squares for water vapor permeability of essential oil-based edible films.

SOV	df	Water Vapor Permeability
Treatments	14	10.03 **
Error	30	2.8557
Total	44	16.2254

** = Highly significant (*p* < 0.01), while df and SOV stand for Degree of Freedom and Source of Variation, respectively.

**Table 7:** Means for water vapor permeability of essential oil-based edible films.

Treatments	Water Vapor Permeability (g.mm/kPa.h.m^2^)
T_1_	3.16 ± 0.07 ^ab^
T_2_	2.60 ± 0.55 ^a^
T_3_	2.20 ± 0.06 ^b–d^
T_4_	1.90 ± 0.04 ^b–e^
T_5_	1.62 ± 0.04 ^c–e^
T_6_	1.23 ± 0.02 ^e^
T_7_	1.91 ± 0.04 ^b–e^
T_8_	1.69 ± 0.06 ^b–e^
T_9_	1.45 ± 0.05 ^de^
T_10_	2.60 ± 0.04 ^ab^
T_11_	1.64 ± 0.06 ^c–e^
T_12_	1.12 ± 0.03 ^e^
T_13_	2.42 ± 0.08 ^a–c^
T_14_	2.24 ± 0.01 ^a–d^
T_15_	1.76 ± 0.04 ^b–e^

Similar letters in the columns indicat non-significant (*p* > 0.05) effect among treatments. T_1_ = Thyme EO 1.0 g, T_2_ = Thyme EO 1.5 g, T_3_ = Thyme EO 2.0 g, T_4_ = Cinnamon EO 1.0 g, T_5_ = Cinnamon EO 1.5 g, T_6_ = Cinnamon EO 2.0 g, T_7_ = Basil EO 1.0 g, T_8_ = Basil EO 1.5 g, T_9_ = Basil EO 2.0 g, T_10_ = Ginger EO 1.0 g, T_11_ = Ginger EO 1.5 g, T_12_ = Ginger EO 2.0 g T_13_ = Cumin EO 1.0 g, T_14_ = Cumin EO 1.5 g, T_15_ = Cumin EO 2.0 g.

**Table 8:** Mean squares for antimicrobial properties of essential oil-based edible films.

SOV	df	*Salmonella* (mm)	*E. coli* (mm)	*S. aureus* (mm)
Treatments	14	59.54 **	142.88 **	44.86 **
Error	30	5.175	2.559	11.520
Total	44	148.959	173.186	252.683

** = Highly significant (*p* < 0.01), while df and SOV stand for Degree of Freedom and Source of Variation, respectively.

**Table 9:** Means for antimicrobial properties of essential oil-based edible films.

Treatments	*Salmonella* (mm)	*E. coli* (mm)	*S. aureus* (mm)
T_1_	10.40 ± 0.6 ^f^	8.50 ± 0.1 ^g^	7.96 ± 0.14 ^d^
T_2_	13.10 ± 0.5 ^c^	9.75 ± 0.25 ^f^	9.24 ± 0.25 ^d^
T_3_	14.60 ± 0.4 ^ab^	12.07 ± 0.3 ^cd^	11.37 ± 0.63 ^cd^
T_4_	8.70 ± 0.3 ^g^	10.30 ± 0.2 ^f^	9.50 ± 0.5 ^ef^
T_5_	10.10 ± 0.2 ^f^	11.86 ± 0.14 ^cd^	11.80 ± 0.6 ^cd^
T_6_	12.60 ± 0.4 ^cd^	14.91 ± 0.2 ^a^	16.10 ± 0.9 ^a^
T_7_	11.80 ± 0.2 ^de^	12.60 ± 0.4 ^c^	13.20 ± 0.4 ^bc^
T_8_	13.40 ± 0.6 ^bc^	13.90 ± 0.3 ^b^	14.30 ± 0.7 ^ab^
T_9_	14.50 ± 0.5 ^ab^	14.70 ± 0.4 ^ab^	15.10 ± 0.9 ^a^
T_10_	10.87 ± 0.63 ^ef^	10.42 ± 0.3 ^ef^	11.20 ± 0.3 ^de^
T_11_	12.48 ± 0.5 ^cd^	12.10 ± 0.2 ^c^	13.07 ± 0.90 ^bc^
T_12_	14.80 ± 0.3 ^a^	15.30 ± 0.2 ^a^	15.22 ± 0.78 ^a^
T_13_	9.90 ± 0.1 ^fg^	11.20 ± 0.3 ^de^	11.70 ± 0.3 ^cd^
T_14_	11.70 ± 0.3 ^de^	12.40 ± 0.4 ^c^	13.20 ± 0.8 ^bc^
T_15_	12.80 ± 0.2 ^cd^	13.93 ± 0.5 ^b^	14.60 ± 0.4 ^ab^

Similar letters in the columns indicate a non-significant (*p* > 0.05) effect among treatments. T_1_ = Thyme EO 1.0 g, T_2_ = Thyme EO 1.5 g, T_3_ = Thyme EO 2.0 g, T_4_ = Cinnamon EO 1.0 g, T_5_ = Cinnamon EO 1.5 g, T_6_ = Cinnamon EO 2.0 g, T_7_ = Basil EO 1.0 g, T_8_ = Basil EO 1.5 g, T_9_ = Basil EO 2.0 g, T_10_ = Ginger EO 1.0 g, T_11_ = Ginger EO 1.5 g, T_12_ = Ginger EO 2.0 g T_13_ = Cumin EO 1.0 g, T_14_ = Cumin EO 1.5 g, T_15_ = Cumin EO 2.0 g.

## Data Availability

The original contributions presented in the study are included in the article, further inquiries can be directed to the corresponding author.
